# Engineering CatM, a LysR-Type Transcriptional Regulator, to Respond Synergistically to Two Effectors

**DOI:** 10.3390/genes10060421

**Published:** 2019-05-31

**Authors:** Melissa P. Tumen-Velasquez, Nicole S. Laniohan, Cory Momany, Ellen L. Neidle

**Affiliations:** 1 Departments of Microbiology, University of Georgia, Athens, GA 30602, USA; dnavgais@uga.edu (M.P.T.-V.); nlanio@uga.edu (N.S.L.); cmomany@uga.edu (C.M.); 2 Pharmaceutical and Biomedical Sciences, University of Georgia, Athens, GA 30602, USA

**Keywords:** LysR, transcription factor, *Acinetobacter*, LTTR, benzoate, muconate, synergism, biosensor

## Abstract

The simultaneous response of one transcriptional regulator to different effectors remains largely unexplored. Nevertheless, such interactions can substantially impact gene expression by rapidly integrating cellular signals and by expanding the range of transcriptional responses. In this study, similarities between paralogs were exploited to engineer novel responses in CatM, a regulator that controls benzoate degradation in *Acinetobacter baylyi* ADP1. One goal was to improve understanding of how its paralog, BenM, activates transcription in response to two compounds (*cis,cis*-muconate and benzoate) at levels significantly greater than with either alone. Despite the overlapping functions of BenM and CatM, which regulate many of the same *ben* and *cat* genes, CatM normally responds only to *cis,cis*-muconate. Using domain swapping and site-directed amino acid replacements, CatM variants were generated and assessed for the ability to activate transcription. To create a variant that responds synergistically to both effectors required alteration of both the effector-binding region and the DNA-binding domain. These studies help define the interconnected roles of protein domains and extend understanding of LysR-type proteins, the largest family of transcriptional regulators in bacteria. Additionally, renewed interest in the modular functionality of transcription factors stems from their potential use as biosensors.

## 1. Introduction

**Figure 1:**
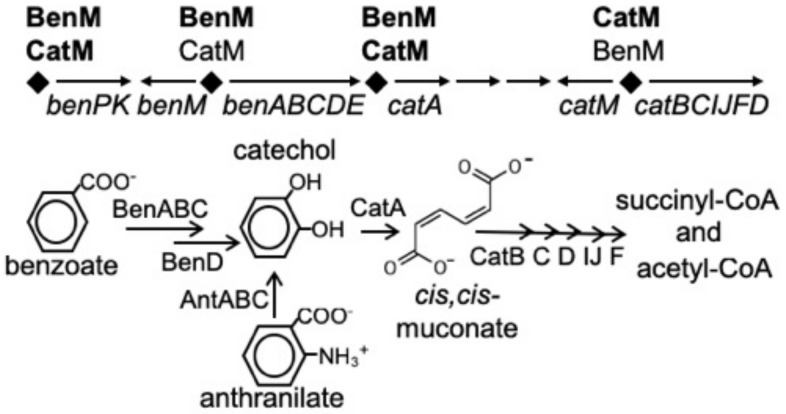
BenM and CatM regulate the *ben* and *cat* genes from four promoter regions (diamonds). The *benABCDE* operon is primarily regulated by BenM from P_benA_, and the *catBCIJFD* operon is primarily regulated by CatM from P_catB_. The encoded enzymes are used for the degradation of benzoate.

Two LysR-type transcriptional regulators (LTTRs) control benzoate degradation by a soil bacterium, *Acinetobacter baylyi* ADP1 [1]. These paralogs, BenM and CatM, have overlapping but distinct functions (Figure 1). BenM controls the initial steps in benzoate consumption by activating transcription of the *benABCDE* operon in response to benzoate and one of its metabolites, *cis,cis*-muconate, hereafter designated muconate [2,3]. CatM, which responds solely to muconate, activates low-level expression from this promoter, P_benA_ [4]. We sought to create a benzoate-responsive CatM that mimics an unusual characteristic of BenM, namely the ability to activate transcription synergistically in response to two effectors. These studies should improve understanding of the molecular basis of this type of transcriptional activation and, in general, facilitate the engineering of LTTRs to respond to novel effectors when designed for varied biotechnology applications.

**Figure 2:**
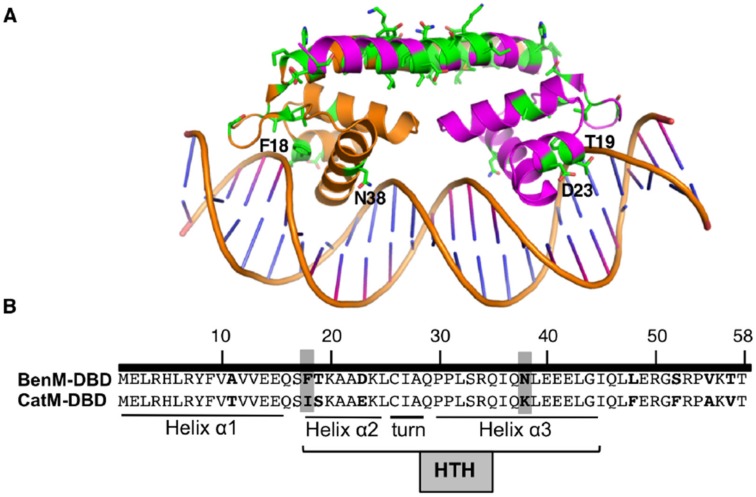
(**A**) Ribbon representation of BenM-DBD and the adjacent “linker” helix bound to DNA (PDB ID 4IHS). Green side chains show differences between BenM and CatM. Labeled residues are discussed in the text. (**B**) Nine amino acids differences in the DBDs (bold), including those at positions 18 and 38 (highlighted). The Helix (α2)-Turn-Helix (α3) (HTH) motif is involved in DNA recognition.

BenM and CatM have similar N-terminal DNA-binding domains (DBDs, Figure 2), and they bind to the same regions of P_benA_ [2,4]. Whereas LTTR-DNA interactions repress basal transcription, conformational changes occur in response to effectors to activate transcription (depicted in Figure S1). Although CatM alone does not activate sufficient transcription for growth on benzoate as the carbon source, mutations can increase CatM-regulated P_benA_ transcription by augmenting its response to muconate or by enabling transcription without an effector [3,4]. Two amino acid replacements in the effector-binding domain (EBD) of CatM each enable BenM-independent growth on benzoate, yet neither of these, nor any other known CatM variant, responds to benzoate.

The structures of BenM-EBD and CatM-EBD are highly conserved (Figure S2) [5,6]. Although benzoate binds in a hydrophobic pocket of BenM, this compound has not been detected in structures of the corresponding region of CatM [5,6]. This pocket is distinct from an inter-domain cleft in BenM and CatM that binds muconate and serves as the typical effector-binding site in LTTRs [6]. The EBDs of LTTRs usually assume the conformation of a periplasmic-binding protein [7,8,9]. However, BenM is the only LTTR known to have a secondary effector-binding site that enables synergistic activation of transcription with different metabolites [2]. In an effort to understand this synergism, we engineered amino acid replacements to make CatM more similar to BenM.

Two residues in the hydrophobic binding pocket of BenM are critical for benzoate-activated transcription, R160 and Y293 [5,6]. When these amino acids are replaced with those at the comparable positions of CatM, H160 and F293, BenM fails to activate transcription in response to benzoate as a sole effector or in combination with muconate [5]. Moreover, benzoate inhibits muconate-activated gene expression in these BenM variants [3,5]. As described here, the converse changes in CatM (H160R and F293Y) did not initially generate a benzoate-responsive CatM. However, efforts to alter CatM were expanded to increase understanding of these representative members of the LTTR family, the largest group of homologous transcriptional regulators in bacteria [10]. After multiple attempts, CatM variants were isolated that respond to benzoate and that activate increased transcription in response to both effectors. As discussed below, changes were required in both the DBD and EBD regions of CatM for this new functionality.

## 2. Materials and Methods

### 2.1. Bacterial Strains and Growth Conditions

*A. baylyi* strains, derived from the wild-type ADP1 [11,12] are listed in Table S1. *Escherichia coli* DH5α (Thermo Fisher Scientific, Waltham, MA, USA) and XL-1 blue (Agilent Technologies, Santa Clara, CA, USA) were used as plasmid hosts. Bacteria were grown on Luria-Bertani (LB) medium at 37 °C [13]. In some cases, *A. baylyi* strains were grown in minimal medium [14,15] with succinate (10 mM), pyruvate (20 mM), benzoate (2 mM), muconate, (2.5 mM), or anthranilate (1.5 mM) as the carbon source. Antibiotics were added when needed at the following final concentrations: ampicillin, 150 μg/mL, kanamycin, 25 μg/mL, spectinomycin, 13 μg/mL, and streptomycin, 13 μg/mL. Growth was monitored by turbidity (OD_600_).

### 2.2. Site-Directed Mutagenesis and Strain Construction by Allelic Replacement

Site-directed mutagenesis of plasmid DNA was conducted with mutagenic primers and methods based on the QuickChange II protocol (Agilent Technologies, Santa Clara, CA, USA [5]). The primers, and the mutations they introduce, are listed in Tables S2 and S3. In some cases, plasmids were constructed using splicing by overlap extension PCR (SOEing) [16]. Linearized plasmid-borne alleles were used to replace chromosomal genes in *A. baylyi* recipient strains by homologous recombination [17,18]. Transformants were identified by phenotypic changes in antibiotic resistance, carbon source utilization or loss of the *sacB* marker (in the presence of 10% sucrose and no NaCl in the medium) [5,18]. The genotypes of mutant strains were confirmed by PCR analysis and DNA sequencing (Genewiz laboratories, South Plainfield, NJ, USA) of the chromosomal regions where changes were introduced.

### 2.3. Selection for BenM-Independent Growth on Benzoate

Strains that form colonies on plates with benzoate as the sole carbon source were defined as Ben^+^. Spontaneous Ben^+^ mutants arising from strains lacking BenM were isolated as described [3,4]. Chromosomal *catM* DNA was recovered from Ben^+^ strains using the gap-repair method [18,19]. Briefly, cells were grown on benzoate medium to mid-log phase, mixed with linearized pBAC184 (Table S2) and plated on LB medium. Transformants with circularized plasmids, resulting from homologous recombination, were selected in medium with ampicillin. Drug-resistant cells were pooled, and plasmid DNA was extracted and used to transform *E. coli*. Recovered *A. baylyi* DNA was tested for the ability to confer a Ben^+^ phenotype to recipient strains (without BenM) by allelic replacement. Mutations were identified by DNA sequencing (Genewiz laboratories, South Plainfield, NJ, USA).

### 2.4. β-Galactosidase (LacZ) Assays

Transcriptional *lacZ* fusions were constructed as described [3,4]. For cultures grown on LB, effectors were added at final concentrations of 500 μM of benzoate or muconate, or when added together, 250 μM of each. Some cultures were grown with pyruvate (20 mM) or muconate (3 mM) as the carbon source. Effectors added to pyruvate-grown cultures were added at the following concentrations: 65 μM benzoate or muconate, or 32.5 μM of each when added together. Growth was measured by optical density (OD_600_), and assays were done when cultures reached late-exponential phase as described [4,5]. Directions from the FlourAce β-galactosidase reporter kit (BioRad, Hercules, CA, USA) were followed. The hydrolysis of the substrate, 4 methylumbelliferyl-galactopyranoside (MUG) to the product 4-methyllumbelliferone (4MU) was detected with a TD-360 miniflourometer (Turner Designs, San Jose, CA, USA). A standard curve was used to quantify 4MU.

### 2.5. Purification of BenM and CatM and Variant Proteins

Plasmids, pBAC433 and pBAC430, were used to express full-length regulators with C-terminal histidine tags, BenM-His and CatM-His, respectively [2]. Plasmids were made to encode variants, pBAC1027 (_BenM-DBD_CatM-His), pBAC1045 (CatM(I18F,K38N)-His), and pBAC1086 (_Ben-DBD_CatM(H160R,F293Y)-His). BenM-His was purified as described [20]. CatM-His and CatM variants were purified similarly but were eluted in a different buffer (30 mM Tris, 500 mM NaCl, 30% glycerol (*v*/*v*), 500 mM imidazole, and 10 mM β-mercaptoethanol (pH 7.9)). Fractions with pure CatM were pooled and dialyzed against a buffer (20 mM Tris-HCl, 250 imidazole (pH 7.9), 500 NaCl, 10% (*v*/*v*) glycerol) to increase solubility, and then were concentrated to 2–10 mg·mL^−1^. Protein concentrations were determined by the method of Bradford with bovine serum albumin as the standard [21]. Proteins fractions were frozen with liquid nitrogen and stored at −70 °C until use.

### 2.6. Electrophoretic Mobility Shift Assays (EMSAs)

Operator-promoter DNA (P_benA_ and P_catB_) was PCR amplified with 5’-6-carboxyfluorescein (6-FAM) labeled primers (Table S3). PCR products, approximately 150–250 bp, were extracted with a gel DNA recovery kit (Zymo Research, Irvine, CA, USA). For EMSAs, 1 nM DNA was incubated with different concentrations of protein (0, 2.5 nM, 5 nM, 10 nM, 20 nM, 40 nM, 80 nM, 160 nM, 320 nM, 640 nM and 1.28 μM) for 1 h at 37 °C with or without muconate, benzoate or both. Effectors in the reaction were present at a total concentration of 1.6 mM individually or at a concentration of 800 μM each when combined. DNA-protein samples were resolved in 6% polyacrylamide gels. Electrophoresis was performed in buffer (40 mM Tris (pH 7.6), 20 mM acetic acid, 1 mM EDTA) for 1 h at 185 volts at 4 °C. When indicated, effectors were added to this buffer at the same concentrations described above. Fluorescently labeled bands were detected using the Amersham Typhoon PhosphorImager system (GE Healthcare Bio-Sciences, Pittsburgh, PA, USA) at 526 nm using the short-pass emission filter. The bound DNA relative to the unbound DNA was quantified by Gel-Pro analyzer (Media Cybernetics, Rockville, MD, USA). Values were fitted into a saturation curve using the equation for one site binding with total accounting for ligand depletion to determine the equilibrium constant (K_d_) (Prism 8 software, GraphPad, San Diego, CA, USA) [22].

### 2.7. Modeling the Transcription Activation Complex at P_benA_ and P_catB_

An atomic model of P_benA_ was created by merging the structures of BenM-DBD bound to DNA [23] and a transcription initiation complex of *E. coli* RNA polymerase (RNAP) [24] using the X3DNA suite [25] with the DNA atoms as alignment guides. Since the Site 1 DNA of P_catB_ has the most sequence identity with *benA* Site 2, the structure of BenM-DBD complexed with P_catB_ Site 1 (PDB ID 4IHS, chains A, B, E, and F) was used to model atoms locally at *benA* Site 2. The structure of BenM-DBD complexed with P_benA_ Site 1 (PDB ID 4IHT, chains A, B, E, and F) was used to model *benA* Site 1. The model for RNAP (PDB ID 4YLN) was positioned assuming that the TTGAAC downstream from P_benA_ Site 2 corresponds to the *E. coli* σ70 binding site with the sequence TTGACA in the 4IHT complex. DNA residues in the three structures were changed and renumbered to match the P_benA_ sequence with the program *mutate_bases*. A composite DNA backbone was generated by calculating the helical parameter sets (shear, stretch, stagger, buckle, prop-tw, opening, shift, slide, rise, tilt, roll, and twist) with program *find_pair* from the three individual DNA double helical segments and then merging the parameters into one file. The two helical parameter sets at the transition regions were averaged. A DNA model was generated from the combined helical parameters using the program *rebuild*. The three atomic structures containing both the DNA and protein residues were aligned on the composite DNA backbone using *align* command of PyMOL [26]. Similar structures of the P_catB_ and P_benA5146_ transcriptional activation complexes were generated by mutating the *benA* DNA sequences in the composite structure. The EBD domains were not fitted to the model because the close proximity of the two binding sites is not consistent with any full-length structure of an LTTR at this time.

## 3. Results

### 3.1. Engineered CatM Variants: Amino Acid Replacements at Positions 160 and 293

In an effort to make CatM respond to benzoate, *A. baylyi* strains were engineered to encode CatM (H160R), CatM (F293Y), or CatM (H160R, F293Y) (designated ACN662, ACN682, and ACN685, Table S1). These amino acid replacements were designed to match residues in BenM-EBD that interact with benzoate (Figure S2). To ensure that transcriptional regulation could be attributable to the CatM variants, *benM* was also disrupted. The engineered strains all grew on anthranilate, catechol, and muconate as sole carbon sources. Such growth requires transcription from the *cat*-operon promoter, P_catB_ (Figure 1), indicating that the variants are functional as *cat*-gene regulators. To assess P_benA_ regulation, growth on benzoate was evaluated. Without BenM, neither wild-type CatM, in strain ISA36 [3] nor CatM (F293Y) in ACN682 supported growth on benzoate as the carbon source. In contrast, CatM (H160R) in ACN662, and CatM (H160R, F293Y), in ACN685, conferred growth on benzoate, suggesting that H160R enables higher than normal levels of P_benA_ transcription.

### 3.2. Transcriptional Regulation of P_benA_ by CatM Variants

**Figure 3:**
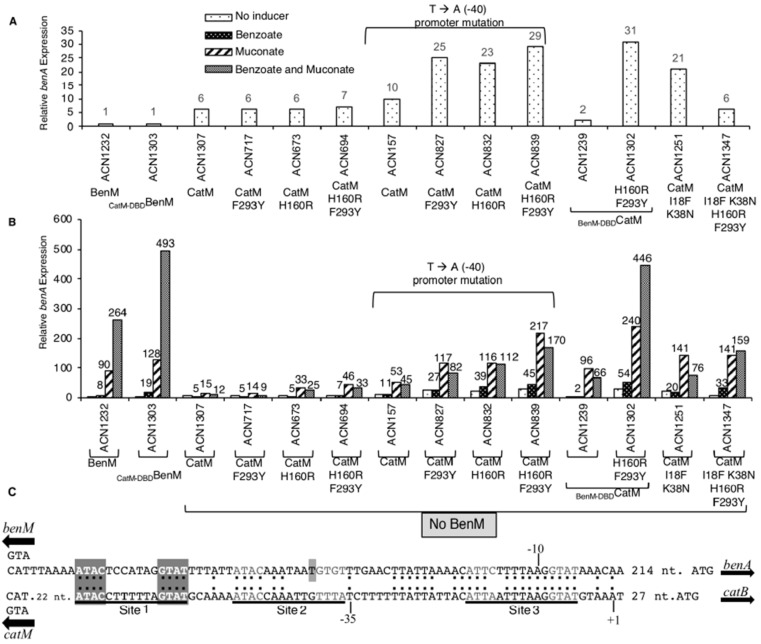
Relative expression of LacZ. Transcription was controlled by P_benA_, or, P_benA5146_, which differs by a mutation at −40 relative to the *benA* transcriptional start site. (**A**) Enlarged scale displays basal activity (no added effectors). (**B**) Effectors were added (or not) as indicated. ACN1232 encodes BenM but not CatM, and ACN1307 encodes CatM but not BenM. Other strains encode a CatM variant. Cultures were grown in LB. LacZ activity is reported relative to uninduced ACN1232 (2.6 ± 0.51 nmol/min/mL/OD600). Activities are averages of at least four repetitions; standard deviations were <20% of the average value. (**C**) Identical nucleotides in aligned P_benA_ and P_catB_ regions are indicated (:). The transcriptional start site (+1) and promoter (−10 and −35 regions) are shown for *catB*. For both regions, Site 1 matches the consensus LTTR-binding motif (T-N_11_-A, within dyad symmetry, ATAC-N_7_-GTAT). Site 2 and Site 3 differ slightly from this consensus. The P_benA5146_ mutation (T) is marked.

A *benA*::*lacZ* fusion was used to replace *benA*, thereby preventing growth on benzoate as the carbon source and enabling benzoate to be tested as a non-metabolized effector [3]. β-Galactosidase (LacZ) activity reflects P_benA_ regulation by CatM(F293Y), CatM(H160R), CatM(H160R,F293Y), or wild-type CatM in strains ACN717, ACN673, ACN694, and ACN1307, respectively (Figure 3). BenM is the major regulator of P_benA_. Without effectors, a tetramer of BenM or CatM, can bind Site 1 and Site 3 to repress transcription of *benA* or *catB* (Figure 3C). Effectors cause a shift in which protein binding to Sites 1 and 2 improves RNAP access to Site 3 [2]. Despite possible cross-regulation, the maximum level of CatM-activated transcription was 18-fold lower than for BenM (ACN1307 versus ACN1232). However, the H160R replacement increased this low-level CatM-mediated response to muconate (ACN673 and ACN694). The elevated transcription levels remained significantly below that for BenM (ACN1232), yet they are comparable to those of other CatM variants that enable BenM-independent growth on benzoate [4]. Regulation by CatM(F293Y), which did not permit growth on benzoate, was comparable to wild-type CatM, which also fails to allow such growth.

While the *catM* changes failed to recapitulate the effects of benzoate with BenM, this failure might be due to problems interacting with P_benA_ DNA as opposed to problems with binding benzoate. In previous studies, CatM activated higher levels of *benA* transcription when there was a transversion (T-to-A) at position −40 relative to the transcriptional start site [3,4]. Here we tested regulation of this promoter (P_benA5146_) by CatM and its variants CatM(F293Y), CatM(H160R), and CatM(H160R,F293Y) using the *lacZ* fusion (in ACN157, ACN827, ACN832, and ACN839, respectively). CatM yielded higher levels of transcription from P_benA5146_ than P_benA_ under all conditions (ACN157 versus ACN1307, Figure 3). At this promoter, the variants all increased transcription even further than wild-type CatM. Benzoate led to expression levels 170% or 155% of the non-induced levels for CatM(H160R) or CatM(H160R,F293Y), respectively (Figure 3). Despite this suggestion of a minor response to benzoate, muconate-mediated transcription was inhibited by benzoate. In contrast, for BenM, benzoate works synergistically with muconate (ACN1232) [2].

### 3.3. Spontaneous Mutant with Increased ben-Gene Expression: Changesne in the CatM-DBD

**Figure 4:**
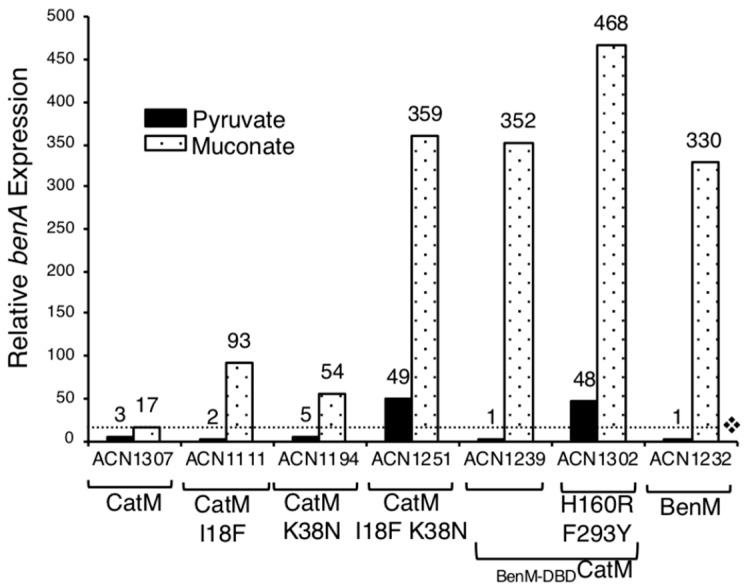
Relative LacZ activity of a chromosomal *benA*::*lacZ* fusion. The only strain encoding BenM is ACN1232, which has no CatM. The dotted line shows the level of the CatM response to muconate (ACN1307). Cultures were grown on pyruvate or muconate as the sole carbon source. Expression is reported relative to the basal level of ACN1232 (6 ± 2 nmol/min/mL/OD600). Values represent the average of at least three independent replicates. Standard deviations were <20% of the average value.

A different approach was used in another attempt to isolate a benzoate-responsive CatM. While ACN682, encoding CatM(F293Y), does not grow on benzoate, its *catM* mutation might facilitate the ability of additional mutations to confer this trait. Based on this rationale, Ben^+^ colonies were directly selected from ACN682, as was done previously for wild-type CatM [3,27]. One ACN682-derived mutant encoded two changes, F293Y and I18F. To test the role of I18F, a strain was made to encode only this change which matches residue 18 of BenM-DBD (Figure 2). CatM(I18F) conferred Ben^+^ growth (ACN1095), indicating increased transcription of P_benA_. When tested with the *benA*::*lacZ* fusion in ACN1111, there was no response to benzoate as a sole effector, and benzoate decreased the muconate-inducible expression (data not shown). It appears that the Ben^+^ phenotype arises from an increase in muconate-induced P_benA_ expression (ACN1111 compared to ACN1307; Figure 4).

Since I18F makes the variant more like BenM, all nine DBD differences were considered (Figure 2). Residue 38, in the recognition helix of the helix-turn-helix (HTH) DNA-binding motif, is implicated in BenM-DNA interactions [23]. Therefore, strains were made to encode CatM(K38N) and CatM(I18F, K38N) (ACN1193 and ACN1249, respectively). Both strains grew on anthranilate, muconate, and benzoate, indicating that the CatM variants are functional. Muconate enabled CatM(K38N) and CatM(I18F, K38N) to activate higher levels of P_benA_ transcription than CatM (ACN1194, ACN1251 versus ACN1307, Figure 4). For the double-replacement variant, gene expression was comparable to that mediated by BenM (in ACN1232). CatM(I18F, K38N) also resulted in high P_benA_ basal expression (Figure 3 and Figure 4). However, there was no transcriptional activation in response to benzoate. Moreover, for both variants with K38N, benzoate decreased the ability of muconate to activate transcription (ACN1251 in Figure 3).

### 3.4. Further Investigation of DBD Residues in BenM and CatM

The ability of BenM to regulate P_benA_ might be weakened by having the residues of CatM at positions 18 and 38. To test this possibility, strains were made to encode BenM(F18I,N38K). When tested with a *benA*::*lacZ* reporter, regulation by this variant was significantly decreased under all conditions relative to BenM. Nevertheless, BenM(F18I,N38K) remained capable of activating transcription synergistically in response to benzoate and muconate (Supplementary Figure S3).

The entire DBD of CatM was replaced with that of BenM by changing nine amino acids. This variant (_BenM-DBD_CatM) enabled growth on muconate, anthranilate, and benzoate as sole carbon sources (ACN1234). When regulating a *benA*::*lacZ* reporter, this variant activated transcription similarly to BenM with muconate (ACN1239 versus ACN1232). However, there was no response to benzoate as the sole effector, and benzoate decreased muconate-inducible expression as observed for the previously studied CatM variants (Figure 3).

### 3.5. Benzoate-Responsive CatM Variants Obtained with Combinations of DBD and EBD Changes

Starting with _BenM-DBD_CatM, we altered the EBD in further attempts to create a response to benzoate. _BenM-DBD_CatM (H160R, F293Y) enabled growth on benzoate (ACN1301, Table 1). This variant (in ACN1302) also enabled muconate or benzoate to activate high-level transcription (Figure 3 and Figure 4). Notably, there was a BenM-like regulatory pattern when benzoate and muconate were added together. This pattern indicated that 11 amino acid changes are sufficient for a benzoate-responsive CatM capable of synergistic transcriptional activation.

Another combination of changes, CatM(I18F,K38N,H160R,F293Y), generated a Ben^+^ strain (ACN1345, Table 1). While two DBD changes enabled muconate-inducible expression, the two additional EBD changes (in ACN1347) were required for induction by benzoate (a 5.5-fold increase in expression, Figure 3). Moreover, when benzoate and muconate were both provided, gene expression increased for CatM(I18F,K38N,H160R,F293Y). In contrast, for CatM(I18F,K38N), benzoate inhibited the response to muconate, Figure 3.

### 3.6. Promoter Specificity and Regulator-DNA Binding Affinities

**Figure 5:**
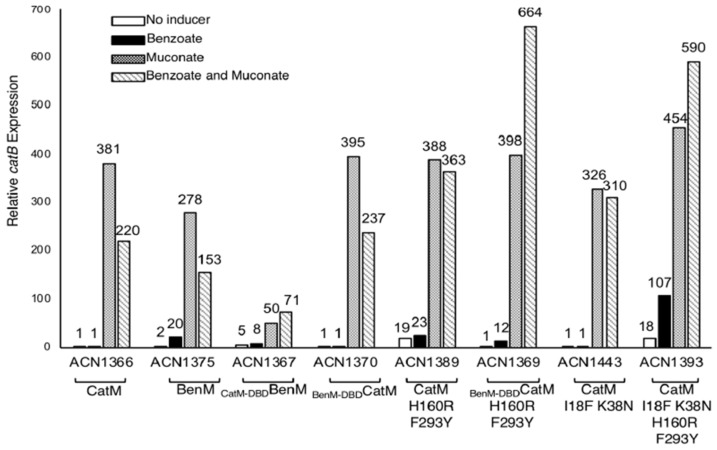
Expression from a chromosomal *catB*::*lacZ* transcriptional reporter. ACN1375 encodes BenM but not CatM. All other strains lack BenM. All have a *benA* disruption to prevent benzoate catabolism. Cultures were grown on 20 mM pyruvate with or without added effectors. β-Galactosidase (LacZ) activity is reported relative to uninduced ACN1366 (0.71 ± 0.5 nmol/min/mL/OD600). Activities are the average of at least three repetitions, and standard deviations were <10% of the average value.

We also studied P_catB_. While CatM-DBD should recognize this region, CatM(H160R,F293Y) did not activate transcription of a *catB*::*lacZ* fusion in response to benzoate (ACN1389, Figure 5). With BenM, P_catB_ transcription was high, but the pattern of effector responses differed from what occurred at P_benA_. Notably, benzoate failed to enhance transcriptional activation in response to muconate (ACN1375, Figure 5). When the BenM-EBD was combined with the CatM-DBD, the overall response to effectors was lessened and the response pattern was altered (ACN1367 compared to ACN1375, Figure 5). As observed at P_benA_, a combination of DBD and EBD changes enabled a response to benzoate. Two CatM variants increased gene expression from P_catB_ in response to benzoate, _BenM-DBD_CatM(F293Y,H160R) in ACN1369 and CatM(I18F,K38N,H160R,F293Y) in ACN1393, Figure 5. With these variants, benzoate enhanced transcriptional activation by muconate.

To evaluate binding to the operator–promoter regions, an electrophoretic mobility gel shift assay (EMSA) was used. However, because the DNA in the assay includes all three regulatory binding sites (Figure 3), the measured K_d_ coefficients do not distinguish between repression and activation. Thus, the affinity of BenM for P_benA_, appears to be of the same order of magnitude regardless of the presence of effectors (Table 2). CatM appeared to have a slightly higher K_d_, which corresponds to a lower affinity, for this DNA. Interestingly, the CatM variants that carry the DBD of BenM have a higher affinity for P_benA_ than either CatM or BenM. This increased affinity appears to require more than the two amino acid changes in the HTH region of the DBD (I18F and K38N). Moreover, increased affinity does not correlate with benzoate responsiveness. These results suggest the hybrid proteins have a conformation that differs from those of the wild-type CatM or BenM.

In experiments with P_catB_, CatM had a higher affinity than BenM for this DNA (Table 2). As for the P_benA_ region, the CatM variants with the entire BenM-DBD region had a higher affinity for the promoter DNA than did the variant with two replacements in the DBD. While it is difficult to infer the significance of the variations in K_d_ values, there appears to be no correlation between the ability of a CatM variant to respond to benzoate and its binding affinity (high or low) for P_benA_ or P_catB_.

### 3.7. Regulator-DNA Interactions at P_benA_: A Structural Model with RNAP

**Figure 6:**
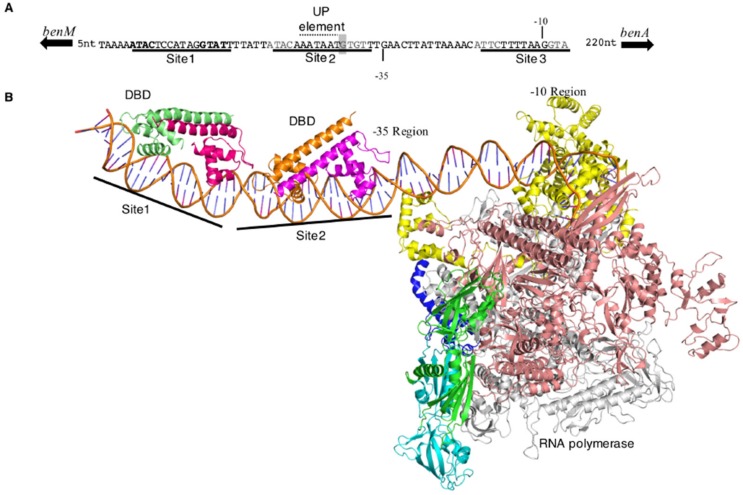
Structural model of BenM-P_benA_ interactions. (**A**). P_benA_ with binding sites for BenM (underlined). A potential UP element [30] is indicated and discussed in the text. The boxed nucleotide (T) corresponds to the mutation in P_benA5146_. (**B**). Model of the initiation complex at P_benA_ rendered as a ribbon representation. BenM-DBD subunits are colored pale green, dark magenta, gold, and magenta going from the 5’ (left) to 3’ of the *benA* promoter; RNA polymerase (RNAP) subunits are green and cyan (α), salmon (β), grey (β’), blue (ω) and yellow (σ).

To understand protein-DNA interactions better, a model was built with available structures of BenM-DBD bound to P_benA_ and P_catB_ and *E. coli* RNAP (Figure 6) [23,24]. In this model, Site 2 overlaps the −35 region of the promoter. A run of adenosine nucleotides within Site 2, with a T nucleotide in the middle, appears to create a promoter feature that may increase transcription by interacting with the RNAP α-subunits, an UP element. The relative position of BenM-DBD to the promoter is typical of a class II σ70 promoter in which a regulator directly contacts domain 4 of the sigma factor of bound RNAP to stabilize the initiation complex [28]. However, the model suggests that the BenM-DBDs would not directly contact σ70. The EBD (not in the model) could make contacts with the sigma factor, but the bulk of the EBD units must sit on the opposite face of the RNAP if BenM is a tetramer. In contrast, for class I σ70 promoters, regulators do contact the α-subunit C-terminal domains (α-CTD), but typically the regulatory proteins bind further upstream of bound RNAP. Protein surface residues of the DBD-dimers that flank the UP-element, such as F31 of GcvA (C26 of BenM), have been implicated in α-CTD interactions [29]. The conserved −35 σ70 recognition-sequence in P_benA_ (TTGAAC vs. consensus TTGACA) suggests that de-repression by conformational change from Site 1 and Site 3 to Site 1 and Site 2 may be a significant aspect of transcriptional activation along with UP-element interactions.

## 4. Discussion

### 4.1. Comparisons of BenM and CatM Provide a Framework to Engineer Effector-Binding Changes in CatM

Residues R160 and Y293 in BenM-EBD, which are critical for benzoate-induced transcriptional activation of P_benA,_ were introduced into CatM. These replacements were designed to create a hydrophobic binding pocket resembling one in BenM that binds benzoate, termed the secondary effector-binding site (Figure S2) [5,6]. Since benzoate decreased the muconate-activated gene expression for these CatM variants (Figure 3), it is likely that benzoate competes with muconate for binding in the primary effector-binding site of this LTTR rather than binding to a secondary site. In CatM(H160R,F293Y) and CatM(H160R), R160 increased the muconate-activated transcription of P_benA_. In BenM, responses to effectors in the primary and secondary binding sites appear to connect through charge-based interactions among three residues that separate muconate and benzoate in the protein structure (R146, E162 and R160) [1].Without benzoate in the secondary binding site of CatM variants, it is unclear how the replacement of H160 with R160 increases the response to muconate. Changes in the local environment may mimic what occurs in BenM upon binding benzoate. Regardless of the mechanism, the increased muconate-responsive transcription due to R160 is consistent with our previous conclusion that this residue in BenM can impact the nearby primary binding site.

The importance of the DBD in P_benA_ regulation was revealed by a spontaneous Ben^+^ mutant. In this mutant, one amino acid change in the HTH region altered transcriptional activation in response to muconate. CatM(I18F), in strain ACN1111, increased muconate-dependent P_benA_ expression more than five-fold relative to CatM, in ACN1307 (Figure 4). A more BenM-like DBD may improve promoter recognition at P_benA_ and thus enable the CatM-EBD to display its typical effector specificity. Some data support the interpretation that DBDs and EBDs follow expected patterns, whereby promoter recognition is governed by the DBD and effector specificity, is controlled by the EBD. For example, replacing the entire DBD of CatM with that of BenM significantly improves regulation of P_benA_ (Figure 3 and Figure 4, ACN1239 compared to ACN1307).

Most results suggest a more complicated relationship among the domains. For example, if the low level of CatM-mediated transcription from P_benA_ were due solely to poor interaction with CatM-DBD, then _CatM-DBD_BenM would be expected to display a typical BenM pattern of effector response but yield low levels of transcription. Instead, the transcriptional levels mediated by this chimeric protein at P_benA_ were higher than for BenM (ACN1303 compared to ACN1232, Figure 3). At P_catB_ this hybrid variant (_CatM-DBD_BenM) might be expected to increase transcriptional activation compared to BenM because CatM is the cognate regulator of *catB*. Instead, the overall expression levels were lowered, and the BenM-mediated pattern of response was altered (ACN1367 compared to ACN1375, Figure 5). Another example that is counter to simple predictions resulted from replacing CatM-DBD with BenM-DBD. This alteration to CatM had little effect at P_catB_ (ACN1366 versus ACN1370, Figure 5). Experimental data are lacking to show if the variant proteins are produced at comparable levels in vivo or whether these proteins are equally stable. Nevertheless, the regulatory patterns, in most cases, are complex. An additional layer of variability results from differences in the flexible helix (the linker helix) that connects the DBD and EBD. In the 30 amino acid residues corresponding to this helix, BenM and CatM are 50% identical and 83% similar in sequence.

### 4.2. Complex Patterns of Regulation

Even without effectors, DBD alterations impacted transcription. Since BenM and CatM repress transcription in the absence of effectors, a several-fold increase in P_benA_ basal expression, which results from the loss of BenM, is interpreted as de-repression [2,3]. Consistent with this model, basal expression in most strains encoding CatM or a CatM variant was approximately 6-fold higher than for the strain encoding BenM (e.g., ACN1307, ACN717, ACN673, or ACN694 compared to ACN1232, Figure 3A). In one notable exception, two replacements in the CatM-DBD, I18F and K38N, caused a 21-fold expression increase in basal expression relative to BenM. This high increase suggests that this variant activates transcription without effectors (ACN1251 compared to ACN1232, Figure 3A). This variant may cause a change in protein structure that relieves binding to Site 3 and helps recruit RNAP. When these two DBD changes were accompanied by changes in the EBD or the DBD, high-level basal expression was no longer observed (ACN1347 and ACN1239, respectively, Figure 3A). Yet when all these EBD and DBD changes were combined in a single variant, _BenM-DBD_CatM(H160R,F293Y), transcriptional activation in the absence of effectors resulted again (ACN1302, Figure 3A). These transcriptional patterns did not correlate meaningfully with changes in the affinity of these proteins for the operator–promoter DNA (Table 2). Better interpretation awaits additional crystal structures and experiments with RNAP. Until then, the crystal structures of BenM-DBD with the Site 1 region of P_benA_ or P_catB_ can provide a framework for considering LTTR-DNA interactions.

### 4.3. Interactions Between DBDs and Operator–Promoter Regions of P_benA_ and P_benA5146_

The I18F replacement in CatM-DBD alters transcription sufficiently to enable Ben^+^ growth. Crystal structures of BenM-DBD-P_benA-Site 1_ and a similar structure, CbnR-DBD-P_cbnA-Site_ indicate that this residue is in helix α2, where it is involved in indirect readout [23,31]. Indirect readout refers to the effects of local nucleotides that cause sequence-dependent deformations of the phosphate backbone to control binding. In BenM, F18 is grouped with residues whose main chain amide N atoms form hydrogen bonds and van der Waals contacts with DNA. The equivalent residue in CbnR, M18, is involved in sugar–phosphate recognition and contributes to DNA-binding strength rather than promoter specificity [31]. CatM binding to P_benA_ is weaker than for BenM (Table 2), which may be due in part from I18 distorting the packing of the helix against the DNA. While binding affinity was not tested for CatM(I18F), the CatM(I18F,K38N) was evaluated (Table 2). Consistent with our interpretation, this variant had an affinity for P_benA_ that was intermediate between that of CatM and BenM. The entire BenM-DBD not only further increased the affinity of CatM variants for P_benA_, but this affinity actually surpassed that of the native BenM (Table 2). These results indicate that binding affinity is not determined solely by the DBD and also suggest there are important variations in the oligomeric conformation of these regulators.

Residue 38 in BenM and CatM is in the recognition helix (α3) of the HTH motif. N38 in BenM can form a hydrogen bond with the DNA phosphate backbone, although this type of non-specific interaction is unlikely to confer specificity for P_benA_. Instead, the importance of this residue may derive from a dynamic interaction with R34, which interacts directly with DNA and provides specificity in DNA recognition. The surrounding residues orient the side chain of R34. An interaction network between these residues and others, including E41 and Q37, might contribute to a conformational switch controlling promoter recognition or RNAP activation at P_benA_ [23]. K38 in CatM would form an electrostatic interaction with the phosphate backbone of P_catB_ and would project deeper into the major grove if a purine were positioned at the interaction point, e.g., nucleotide A36 in the BenM-DBD-*catB* DNA structure. However, the methyl-group of thymine, as found in this nucleotide position of P_benA_, would impede this direct interaction. Consistent with this possibility, CatM(K38N) increased muconate-activated transcription of P_benA_ better than the wild-type CatM (Figure 4). Similar interactions are observed in other LTTRs, such as CbnR (A38, R34, and D42) [31], MetR (S38, S34, H35, and Q42) [32], DntR (R43, N39, T46 and A47) [33] and Tsar (Q38, D42, and S34) [34].

DNA interactions with residue 38 in the DBD may influence effects at P_benA5146_. Binding at Site 2 of this promoter (which has a T-to-A transversion) by CatM will be enhanced compared to P_benA_ due to K38 interactions with the partial negative charge on the N7 atom of the adenine base. Interactions of BenM with P_benMA5146_ may not be negatively impacted because a water molecule can bridge to the N7. Thus, the interaction of CatM with P_benMA5146_ may be stabilized at Site 2 but not Site 3, thereby disrupting the equilibrium between the protein binding to Site 2 (stabilized by effectors) and Site 3 (stabilized by lack of effectors). This alteration could result in the de-repression of basal activity observed at this promoter for CatM and help explain transcriptional activation in the absence of effectors for the CatM variants with F293Y and/or H160R replacements in the CatM-EBD (Figure 3A).

### 4.4. Interactions Between DBDs and Operator–Promoter Regions of P_catB_

Factors affecting K38 interactions with P_benA5146_ may also affect recognition at P_catB_. Between the two half sites of dyad symmetry in P_catB_ Site 2, a G corresponds to the position of the T-to-A mutation of P_benMA5146_ (Figure 3C). In general, the sequence ATAC-pyrimidine-N_5_-purine-GTAT will favor binding at P_catB_ by CatM. This pattern is observed for Sites 1 and 2. At P_benA_, Site 2 swaps the arrangement of the purine/pyrimidine pair, a situation that may partially explain why wild-type CatM shows significantly reduced responses at P_benA_.

BenM activated transcription relatively strongly at P_catB_ despite a different pattern of response to benzoate compared to its activity at P_benA_ (Figure 5). As measured by EMSAs, the affinity of BenM for P_benA_ and P_catB_ is comparable. While the native CatM has a higher affinity for its cognate P_catB_ promoter than for P_benA_, all proteins with a native BenM-DBD have a comparable affinity for both promoters (Table 2). It is not yet possible to discern the features that control such variation in binding and transcriptional control, and a better understanding of LTTR interactions with RNAP is needed.

### 4.5. A Model of P_benA_ and P_catB_ Promoters with RNAP and the DBDs of BenM and CatM

Aspects of the model described above for protein interactions with P_benA_ also apply to P_catB_. However, some important differences stem from DNA sequence variation. P_catB_ differs in the region of the 3’ half-site of symmetry in Site 2 from that of P_benA_. The sequence of P_catB_ in this region (TCTTTT, Figure 3) does not match the −35 consensus sequence of σ70 promoters. Further, the 3’ half-site of Site 2 (TTTA) lacks similarity to the canonical binding 3’ half-site shared by BenM and CatM, GTAT (P_benA_, GTGT). This sequence divergence suggests that the regulators at P_catB_ may play a more direct role in transcription initiation by replacing the -35 region DNA interactions with σ70 with compensating LTTR-RNAP contact. Analysis of the electrostatic surfaces of σ70 and BenM-DBD at the *benA* promoter show a strongly negative surface created by D23 in BenM (E23 in CatM) and a strongly positive surface defined by residues K593, R596, and K597 of σ70 which are part of the conserved regulatory region 4.2 of the sigma factor. Clearly, rotation of the RNAP holoenzyme toward BenM by moving forward a few base pairs along P_catB_ will position the RNAP against BenM or CatM in a complementary fashion. The amino acids in this region 4 of sigma have been mapped as binding residues in other sigma regulatory molecules [35]. Between the half-sites of symmetry of Site 2 in P_catB,_ there is a mix of A and T nucleotides that could act as an UP-element to stabilize αCTD interactions, as observed in P_benA_. This combination of σ70 and αCTD interactions would make the P_catB_ a mixed class I–class II promoter. However, the UP-element sequence differs from that of P_benA_ because of the flanking C/G nucleotide pair at the 5’ half-site of Site 2. Alternatively, if CatM and BenM bind only to the 5’ half-site sequence, the 3’ area of Site 2 might be occupied by σ70 (perhaps at the intervening TTGTT) and allow some conformational flexibility for the previously mentioned electrostatic interactions to be favored.

### 4.6. Broader Implications and Conclusions

Although LTTRs have been studied for more than 30 years, many aspects of their structure and function remain unclear [8]. Because of their similarities and the overlap in their control of a complex regulon, BenM and CatM provide unique opportunities for comparative investigation [1]. Furthermore, their role in aromatic compound catabolism holds promise for biotechnology applications, including lignin valorization [18]. Because of such applications, these and related regulators are receiving renewed attention. For example, transcriptional regulators that respond to aromatic compounds are useful as biosensors [36]. In a recent study, high throughput methods were developed to combine protein domains for DNA binding and those for effector responses with the aim of creating benzoate-responsive biosensors [37]. This approach builds on the modular nature of bacterial transcriptional regulators.

Our studies demonstrate that regulatory alterations can be engineered to integrate multiple signals by enabling the simultaneous response to more than one effector molecule. Although it took repeated efforts, we obtained CatM variants that recognize and respond to benzoate. In some variants, benzoate increased the transcriptional response to muconate, thereby approximating the synergistic transcriptional regulation mediated by BenM. This type of rapid signal amplification has potential uses in metabolic engineering and synthetic biology. While it is not yet possible to interpret some characteristics of the variant regulators that were studied, our results lay the foundation for continued investigation. LTTRs are more than the sum of their parts; both the DBDs and the EBDs affected promoter specificity as well as transcriptional responses to effectors. The abundance of this family of regulators and the importance of LTTR-regulated processes underscore the value of continued research.

## Figures and Tables

**Table 1:** Growth on Benzoate as the Sole Carbon Source ^a^.

Strain	Relevant Characteristics	Generation Time (min) ^b^	Lag Time (h) ^c^
ADP1	Wild-type	70 ± 5	4.5 ± 1
ISA36	No BenM	No growth	No growth
ACN682	No BenM, CatM(F293Y)	No growth	No growth
ACN1095	No BenM, CatM(I18F)	175 ± 3	18 ± 3
ACN1193	No BenM, CatM(K38N)	145 ± 6	11 ± 1
ACN1249	No BenM, CatM(I18F,K38N)	85 ± 2	5.5 ± 2
ACN1234	No BenM, _BenM-DBD_CatM	82 ± 3	5 ± 1.5
ACN1301	No BenM, _BenM-DBD_CatM(F293Y,H160R)	81 ± 4	5 ± 1
ACN1345	No BenM, CatM(I18F,K38N,H160R,F293Y)	83 ± 3	5 ± 1.5
ACN1294	_CatM-DBD_BenM, No CatM	186 ± 6	21 ± 3

^a^ Strains had comparable growth rates with succinate as the sole carbon source (data not shown); ^b^ Averages of at least four determinations; ^c^ Time between inoculation and start of exponential growth.

**Table 2:** Estimated Binding Affinities of Regulators to DNA With and Without Effectors ^a^.

DNA	Transcriptional Regulator	Effector Added to Protein-DNA Complex			
		No Effector K_d_ (nM)	Benzoate K_d_ (nM)	Muconate K_d_ (nM)	Muconate and Benzoate K_d_ (nM)
**P_benA_**	Wild-type BenM	32 ± 3	17 ± 2	41 ± 3	46 ± 3
	Wild-type CatM	70 ± 3	71 ± 4	67 ± 3	71 ± 4
	_BenM-DBD_CatM	2 ± 0.5	4 ± 1	3 ± 0.5	9 ± 3
	_BenM-DBD_CatM(F293Y,H160R)	1 ± 0.2	4 ± 1	2 ± 0.5	4 ± 2
	CatM(18F,K38N)	42 ± 2	32 ± 3	34 ± 1	32 ± 4
**P_catB_**	Wild-type BenM	26 ± 3	30 ± 2	16 ± 2	19 ± 1
	Wild-type CatM	11 ± 2	19 ± 3	10 ± 1	19 ± 2
	_BenM-DBD_CatM	1 ± 0.2	3 ± 0.5	3 ± 0.2	4 ± 1
	_BenM-DBD_CatM(F293Y,H160R)	3 ± 0.5	3 ± 0.5	3 ± 0.5	3 ± 0.5
	CatM(18F,K38N)	28 ± 2	13 ± 2	8 ± 2	14 ± 0.5

^a^ Values represent averages of four replicates with *p* > 0.001.

## Supplementary Materials

The following are available online at https://www.mdpi.com/2073-4425/10/6/421/s1, Figure S1. Regulatory model for P_benA_. Figure S2. Structural comparisons of BenM-EBD and CatM-EBD. Figure S3. Changes in BenM-DBD affect relative *benA* expression. Table S1. *Acinetobacter baylyi* strains. Table S2. Plasmids. Table S3. Primers.
