# Supplementary material for: Engineering CatM, a LysR-Type Transcriptional Regulator, to Respond Synergistically to Two Effectors
Source: Genes (Basel). 2019 May 31;10(6):421. doi: 10.3390/genes10060421 (PMC6628147; doi:10.3390/genes10060421)
Supplement: Supplementary file 1 [file genes-10-00421-s001.pdf]

Supplementary Information For:

Engineering CatM, a LysR-type transcriptional regulator, to respond synergistically to two effectors

Melissa P. Tumen-Velasquez[1], Nicole S. Laniohan[1,2], Cory Momany[1,2] and Ellen L. Neidle[1,*]

[1] Departments of Microbiology and [2]Pharmaceutical and Biomedical Sciences, University of Georgia, Athens, GA 30602, USA; dnavgais@uga.edu (M.P.T.-V.); nlanio@uga.edu (N.S.L.); cmomany@uga.edu (C.M.)
* Correspondence: eneidle@uga.edu; Tel.: +1-706-542-2852

This file includes:

Figures

Figure S1. Regulatory model for P_{benA}
Figure S2. Structural comparisons of BenM-EBD and CatM-EBD
Figure S3. Changes in BenM-DBD affect relative *benA* expression

Tables

Table S1. *Acinetobacter baylyi* strains
Table S2. Plasmids
Table S3. Primers

Supplementary Figure S1

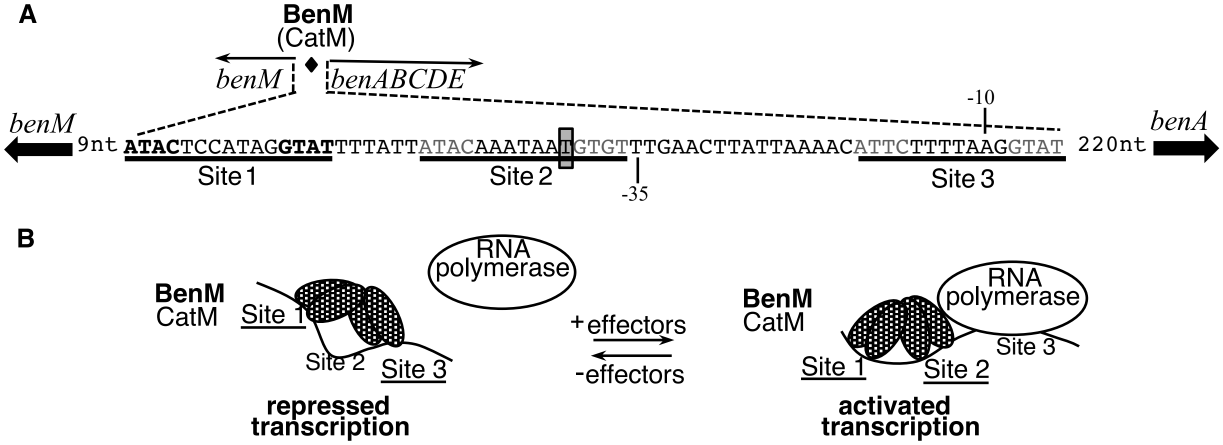

Figure S1. Regulatory model for P_{benA}. While BenM is the primary transcriptional regulator of the *benABCDE* operon, CatM plays a minor role at this region and binds to the same sites as BenM [2,3]. (A) The three underlined binding sites for BenM and CatM each consist of a sequence with dyad symmetry that can interact with the DBD regions of two subunits of a regulatory homotetramer. The perfect dyad symmetry of Site 1 (ATAC-N_7-GTAT) matches the consensus for an LTTR-binding motif (T-N_{11}-A within a region of two half sites of dyad symmetry). In Site 2 and Site 3 a one nucleotide mismatch to the consensus sequence reduces the dyad symmetry. The -10 and -35 regions of the *benA* promoter (P_{benA}) are indicated. BenM is negatively autoregulated [2]. (B) In the absence of effectors BenM binds Site 1 and Site 3 to repress transription from P_{benA}. Effectors (benzoate and/or muconate) can alter the protein conformation to shift the position of the tetramer towards binding Site 1 and Site 2 to activate transcription [2]. The model for CatM-mediated P_{benA} regulation is similar, but CatM activates transcription only in response to muconate [7].

Supplementary Figure S2

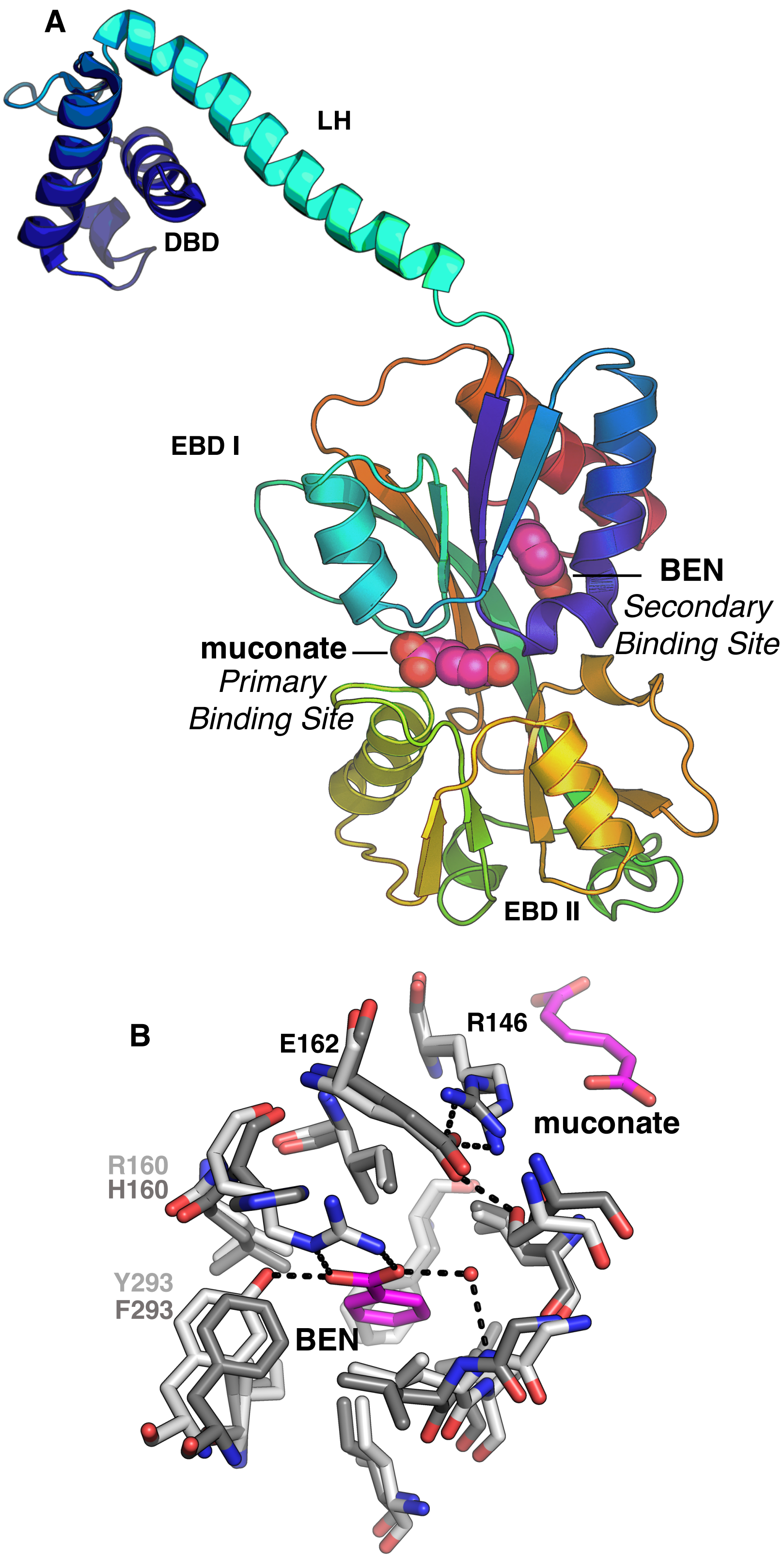

Figure S2. Structural comparisons of BenM-EBD and CatM-EBD. (A) The structure of one full-length subunit of BenM [1] illustrates the domains and organization of a typical LTTR protein in which the N-terminal DNA-binding domain (DBD) is connected by a linker helix (LH) to an effector-binding domain (EBD), made up of two subdomains, EBD I and EBD II. The EBDs of LTTRs assume the conformation of periplasmic-binding proteins [4]. A cleft between the two EBD subdomains forms the typical effector-binding site in an LTTR, designated as the primary binding site [5,6]. In BenM and CatM, muconate binds in this primary binding site. In BenM, a secondary binding site exists where benzoate (BEN) can bind. Effectors are shown in space-filling representation [3,5]. (B) The BenM-EBD structure (light grey) shows BEN interacting with residues Y293 and R160 and illustrates the relative positions of the two effectors. E162 and R146 appear to contribute to the synergistic response to BEN and muconate [5]. In the structure of CatM-EBD (dark grey), F293 and H160 replace the corresponding F and R residues of BenM-EBD.

Supplementary Figure S3

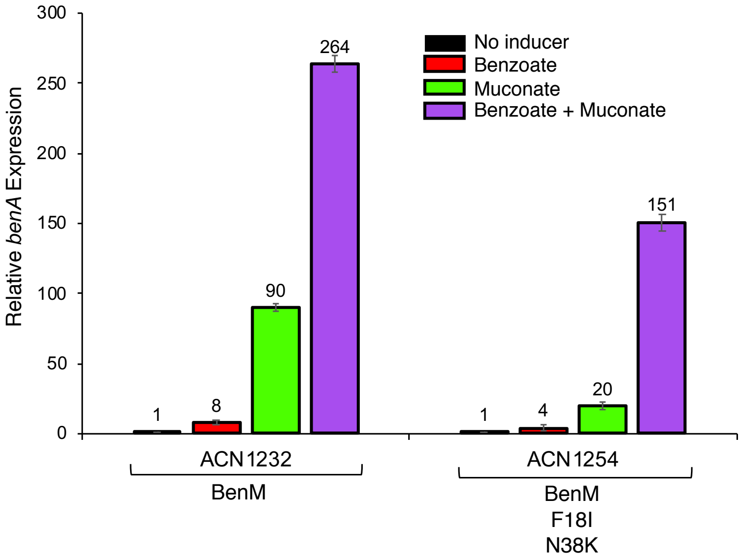

Figure 4. Changes in BenM-DBD affect relative *benA* expression. Two amino acid replacements in the DBD of BenM (encoded by ACN1254) were engineered to match residues found in CatM at positions 18 and 38. Regulation of a chromosomal *benA*::*lacZ* fusion by the variant, BenM(F18I,N38K), was compared with that by wild-type BenM (encoded by ACN1232). Neither strain encodes CatM. Cultures were grown on LB and effectors were added (or not) as indicated. LacZ activity is reported relative to uninduced ACN1232 (2.6 ± 0.51 nmol/min/mL/OD600). Activities are averages of at least four repetitions; standard deviations were <20% of the average value.

Supplementary Table S1.

Table S1. *Acinetobacter baylyi* strains[a]

A. baylyi Strain	Relevant characteristics[b,c]	Source
ADP1	Wild type (BD413)	[8,9]
ISA13	*catM*::ΩS*4013*	[10]
ISA36	*benM*:: ΩS*4036*	[11]
ACN32	*benA*::*lacZ*-Km[R]*5032*	[11]
ACN146	*benM*::ΩS*4036*; *benMA5146* (point mutation T →A in P_{benA})	[11]
ACN157	*benM*::ΩS*4036*; *benA*::*lacZ*-Km[R]*5032*; *benMA5146*	[11]
ACN613	*catM*::*sacB*-Km[R]*5613*<br>pBAC708/AlwNI X ADP1; selected by Km[R]	This study
ACN614	*benM*::ΩS*4036*; *catM*::*sacB*-Km[R]*5613*<br>ISA36 lysate DNA X ACN613; selected by SpSm[R]	This study
ACN637	*benM*::*sacB*-Km[R]*5624*	[12]
ACN662	*benM*::ΩS*4036*; *catM5662* [CatM(H160R)]<br>pBAC1109/AatII X ACN614; selected in the presence of sucrose	This study
ACN673	*benM*::ΩS*4036*; *benA*::*lacZ*-Km[R]*5032*; *catM5662* [CatM(H160R)]<br>pBAC54/XmnI X ACN662; selected by Km[R]	This study
ACN682	*benM*::ΩS*4036*; *catM5682* [CatM(F293Y)]<br>pBAC1108/AatII X ACN614; selected in the presence of sucrose	This study
ACN685	*benM*::ΩS*4036*; *catM5685* [CatM(H160R,F293Y)]<br>pBAC1140/AatII X ACN614; selected in the presence of sucrose	This study
ACN694	*benM*::ΩS*4036*; *benA*::*lacZ*-Km[R]*5032*; *catM5685* [CatM(H160R,F293Y)]<br>pBAC54/XmnI X ACN685; selected by Km[R]	This study
ACN717	*benM*::ΩS*4036*; *benA*::*lacZ*-Km[R]*5032*; *catM5682* [CatM(F293Y)]<br>pBAC54/XmnI X ACN682; selected by Km[R]	This study
ACN737	*benM*::ΩS*4036*; *catM5737* [CatM(F293Y, I18F)]; Spontaneous Ben[+] mutant derived from ACN682	This study
ACN821	*benM*::ΩS*4036*; *benMA5146*; *catM*::*sacB*-Km[R]*5613*<br>pBAC708/AlwNI X ACN146; selected by Km[R]	This study
ACN822	*benM*::ΩS*4036*; *benMA5146*; *catM5662* [CatM(H160R)]<br>pBAC1109/AatII X ACN821; selected in the presence of sucrose	This study
ACN823	*benM*::ΩS*4036*; *benMA5146*; *catM5682* [CatM(F293Y)]<br>pBAC1108/AatII X ACN821; selected in the presence of sucrose	This study
ACN827	*benM*::ΩS*4036*; *benMA5146*; *benA*::*lacZ*-Km[R]*5032*; *catM5682* [CatM(F293Y)]<br>pBAC54/XmnI X ACN823; selected by Km[R]	This study
ACN831	*benM*::ΩS*4036*; *benMA5146*; *catM5685* [CatM(H160R,F293Y)]<br>pBAC1140/AatII X ACN821; selected in the presence of sucrose	This study
ACN832	*benM*::ΩS*4036*; *benMA5146*; *benA*::*lacZ*-Km[R]*5032*; *catM5662* [CatM(H160R)]<br>pBAC54/XmnI X ACN822; selected by Km[R]	This study
ACN839	*benM*::ΩS*4036*; *benMA5146*; *benA*::*lacZ*-Km[R]*5032*; *catM5685*[CatM(H160R,F293Y)]<br>pBAC54/XmnI X ACN831; selected by Km[R]	This study
ACN843	Δ*benM5389*; *catM*::*sacB*-Km[R]*5613*	[13]
ACN1090	Δ*catM51090*<br>pBAC887/AatII X ACN613; selected in the presence of sucrose	This study

Table S1. continued

A. baylyi Strain	Relevant characteristics[b,c]	Source
ACN1095	*benM*::ΩS4036; *catM51095* [CatM(I18F)]<br>pBAC938/AatII X ISA36; selected by growth on benzoate, Ben$^+$	This study
ACN1111	*benM*::ΩS4036; *benA*::*lacZ*-Km^R*5032*; *catM51095* [CatM(I18F)]<br>pBAC54/XmnI X ACN1095; selected by Km^R	This study
ACN1150	*benM*::ΩS4036; *catM51095* [CatM(I18F)]; *catB*::*lacZ*-Km^R*5534*<br>pBAC675/KpnI X ACN1095; selected by Km^R	This study
ACN1193	*benM*::ΩS4036; *catM51188* [CatM(K38N)]<br>pBAC961/AatII X ISA36; selected by growth on benzoate, Ben$^+$	This study
ACN1194	*benM*::ΩS4036; *benA*::*lacZ*-Km^R*5032*; *catM51188* [CatM(K38N)]<br>pBAC54/XmnI X ACN1193; selected by Km^R	This study
ACN1198	*benM*::ΩS4036; *catM51188* [CatM(K38N)]; *catB*::*lacZ*-Km^R*5534*<br>pBAC675/KpnI X ACN1193; selected by Km^R	This study
ACN1232	*benA*::*lacZ*-Km^R*5032*; *catM*::ΩS4013<br>pBAC54/XmnI X ISA13; selected by Km^R	This study
ACN1234	Δ*benM5389*; *catM51234*; [$_{\text{BenM-DBD}}$CatM]; allele replaces wild-type *catM*<br>pBAC1025/AatII X ACN843; selected in the presence of sucrose	This study
ACN1237	Δ*benM5389*; *catM51234* [$_{\text{BenM-DBD}}$CatM]; *catB*::*lacZ*-Km^R*5534*<br>pBAC675/KpnI X ACN1234; selected by Km^R	This study
ACN1238	Δ*catM51090*; *benM*::*sacB*-Km^R*5624*<br>pBAC709/AlwNI X ACN1090; selected by Km^R	This study
ACN1239	Δ*benM5389*; *benA*::*lacZ*-Km^R*5032*; *catM51234* [$_{\text{BenM-DBD}}$CatM]<br>pBAC54/XmnI X ACN1234; selected by Km^R	This study
ACN1240	*benM51240*; Δ*catM51090*; *benM51240;* [BenM(F18I)]<br>pBAC936/AatII X ACN1238; selected in the presence of sucrose	This study
ACN1249	Δ*benM5389*; *catM51249* [CatM(I18F,K38N)]<br>pBAC1040/AatII X ACN843; selected in the presence of sucrose	This study
ACN1250	Δ*catM51090*; *benM51250* [BenM(F18I,N38K)]<br>pBAC1041/AatII X ACN1238; selected in the presence of sucrose	This study
ACN1251	Δ*benM5389*; *benA*::*lacZ*-Km^R*5032*; *catM51249* [CatM(I18F,K38N)]<br>pBAC54/XmnI X ACN1249; selected by Km^R	This study
ACN1252	Δ*benM5389*, *catM51249* [CatM(I18F,K38N)], *catB*::*lacZ*-Km^R*5534*<br>pBAC675/KpnI X ACN1249; selected by Km^R	This study
ACN1254	Δ*catM51090*; *benM51250* [BenM(F18I,N38K)]; *benA*::*lacZ*-Km^R*5032*<br>pBAC54/XmnI X ACN1250; selected by Km^R	This study
ACN1264	Δ*catM51090*<br>pBAC949/AatII X ACN1238	This study
ACN1266	Δ*catM51090*; *catB*::*lacZ*-Km^R*5534*<br>pBAC675/KpnI X ACN1264; selected by Km^R	This study
ACN1293	Δ*benM5389*<br>pBAC945/AatII X ACN843; selected in the presence of sucrose	This study
ACN1294	Δ*catM51090*; *benM51294* [$_{\text{CatM-DBD}}$BenM]; allele replaces wild-type *benM*<br>pBAC1074/AatII X ACN1238; selected in the presence of sucrose	This study

Table S1. continued

A. baylyi Strain	Relevant characteristics[b,c]	Source
ACN1301	Δ*benM5389*; *catM51301* [BenM-DBDCatM(H160R,F293Y)]; allele replaces wild-type *catM* pBAC1078/AatII X ACN843; selected in the presence of sucrose	This study
ACN1302	Δ*benM5389*; *benA*::*lacZ*-Km^R*5032*; *catM51301* [BenM-DBDCatM(H160R,F293Y)] pBAC54/XmnI X ACN1301; selected by Km^R	This study
ACN1304	*benM51294*; Δ*catM51090*; *catB*::*lacZ*-Km^R*5534* pBAC675/KpnI X ACN1294; selected by Km^R	This study
ACN1307	Δ*benM5389*; *benA*::*lacZ*-Km^R*5032*; *catM51293* pBAC54/XmnI X ACN1293; selected by Km^R	This study
ACN1308	Δ*benM5389*; *catM51293*; *catB*::*lacZ*-Km^R*5534* pBAC675/KpnI X ACN1293; selected by Km^R	This study
ACN1344	Δ*benM5389*; *catM51344* [CatM(I18F,K38N,H160R,F293Y)] pBAC1071/AatII X ACN843; selected in the presence of sucrose	This study
ACN1347	Δ*benM5389*; *benA*::*lacZ*-Km^R*5032*; *catM51344* [CatM(I18F,K38N,H160R,F293Y)] pBAC54/XmnI X ACN1344; selected by Km^R	This study
ACN1359	Δ*benM5389*; *catM51301* [BenM-DBDCatM(H160R, F293Y)]; *catB*::*lacZ*-Km^R*5534* pBAC675/KpnI X ACN1301; selected by Km^R	This study
ACN1366	Δ*benM5389*; *benA*::Ω*S51366*; *catB*::*lacZ*-Km^R*5534* pBAC393/AatII X ACN1308; selected by SpSm^R	This study
ACN1367	Δ*catM51090*; *benA*::Ω*S51366*; *benM51294* [CatM-DBDBenM]; *catB*::*lacZ*-Km^R*5534* pBAC393/AatII X ACN1304; selected by SpSm^R	This study
ACN1369	Δ*benM5389*; *benA*::Ω*S51366*; *catM51301* [BenM-DBDCatM(H160R, F293Y)]; *catB*::*lacZ*-Km^R*5534* pBAC393/AatII X ACN1359; selected by SpSm^R	This study
ACN1370	Δ*benM5389*; *benA*::Ω*S51366*; *catM51234* [BenM-DBDCatM]; *catB*::*lacZ*-Km^R*5534* pBAC393/AatII X ACN1237; selected by SpSm^R	This study
ACN1375	*benA*::Ω*S51366*; ΔcatM*51090*; *catB*::*lacZ*-Km^R*5534* pBAC393/AatII X ACN1266; selected by SpSm^R	This study
ACN1381	Δ*benM5389*; *catM5662* [CatM(H160R,F293Y)] pBAC1140/AatII X ACN843; selected in the presence of sucrose	This study
ACN1383	Δ*benM5389*; *catM5662* [CatM(H160R,F293Y)]; *catB*::*lacZ*-Km^R*5534* pBAC675/KpnI X ACN1381; selected by Km^R	This study
ACN1389	Δ*benM5389*; *benA*::Ω*S51366*; *catM5662* [CatM(H160R,F293Y)]; *catB*::*lacZ*-Km^R*5534* pBAC393/AatII X ACN1383; selected by SpSm^R	This study
ACN1390	Δ*benM5389*; *catM51344* [CatM(I18F,K38N,H160R,F293Y)]; *catB*::*lacZ*-Km^R*5534* pBAC675/KpnI X ACN1344; selected by Km^R	This study
ACN1393	Δ*benM5389*; *benA*::Ω*S51366*; *catM51344* [CatM(I18F,K38N,H160R,F293Y)]; *catB*::*lacZ*-Km^R*5534* pBAC393/AatII X ACN1390; selected with sucrose	This study
ACN1443	Δ*benM5389*; *benA*::ΩSm^R*51366*; *catM51249* [CatM(I18F,K38N)]; *catB*::*lacZ*-Km^R*5534* pBAC393/AatII X ACN1252; selected by SpSm^R	This study

[a] Srains were derived from ADP1, previously known as *Acinetobacter calcoaceticus* or *Acinetobacter sp.* [9]

[b] For strains made by allelic replacement, blue text shows the DNA that transformed (X) the recipient strain. DNA was either linearized plasmid (pBAC number/enzyme used to linearize the plasmid) or a cell-free lysate containing genomic DNA of the donor.

[c] ΩS and ΩK are omega cassettes for SpSm^R from pUI1638 (or pHP45) and Km^R from pUI1637 [14,15].

Supplementary Table S2.

Table S2. Plasmids

Plasmid	Relevant characteristics[a]	Source
pUC18	Ap^R; cloning vector	[16]
pUC19	Ap^R; cloning vector	[16]
pHP45	Source of ΩS drug-resistance cassette, Sp^{R}Sm^R	[15]
pRMJ1	Source of *sacB*-Km^R cassette	[17]
pUI1638	Source of ΩS drug-resistance cassette, Sp^{R}Sm^R	[14]
pET-21b	Ap^R; T7 expression vector	Novagen
pKOK6	Ap^R Km^R; source of promoterless *lacZ*::Km^R cassette	[18]
pIB1	Ap^R; partial *cat* region (1,443,167-1,449,480)[b]	[19]
pIB3	Ap^R; partial *catM* (1,441,385-1,444,456)[b]	[19]
pIB101	Ap^R SmSp^R; ΩS was excised from pHP45 as a XmaI fragment and ligated to pIB1351 digested with BspEI (1,435,092)[b]	This study
pIB1351	Ap^R; partial *ben* region (1,432,125-1,437,224)[b]	[20]
pIGG5	Ap^R; partial *ben* region (1,432,125-1,433,495)[b]	[11]
pIGG13	Ap^R; partial *ben* region with an internal KpnI deletion (1,432,125-1,439,437)[b]. This plasmid, linearized with KpnI, allows capture of the *A. baylyi* chromosomal region containing *benM* using the gap-repair method [21]	This study
pBAC7	Ap^R; *benKM* (1,432,124-1,434,525)[b] region in pUC19	[12]
pBAC54	Ap^R Km^R; *lacZ*-Km^R cassette in NsiI site (1,435,326)[b] in *benA* (*ben* region 1,433,877-1,437,224)[b] in pUC19	[11]
pBAC184	Ap^R; partial *cat* region with internal ClaI deletion (1,442,211-1,447,468)[b]. This plasmid, linearized with ClaI, allows capture of the *A. baylyi* chromosomal region containing *catM* using the gap-repair method [21]	[13]
pBAC393	Ap^R SmSp^R; *benA*ΩS was excised from pIB101 as a NsiI fragment and ligated to PstI-digested pIGG5	This study
pBAC430	Ap^R; *catM* (1,443,682-1,444,590)[b] in pET-21b	[2]
pBAC433	Ap$^{R;}$ *benM* (1,443,017-1,433,928)[b] in pET-21b	[2]
pBAC675	Ap^R Km^R; *catB* (1,444,770-1,445,789)[b]; *lacZ*-Km^R *catIJF* (1,447,225-1,449,044)[b] in pUC19	[7]
pBAC708	Ap^R Km^R; *sacB*-Km^R cassette inserted in *catM* in pUC19. ADP1 DNA surrounds cassette (1,443,514-1,444,252, upstream of *sacB*) and (1,444,252-1,444,461 adjacent to the Km^R marker)	[13]
pBAC709	Ap^R Km^R; *benKM* region (1,432,128-1,433,880)[b] in pUC19. Contains *sacB*-Km^R cassette in SalI site (1,433,494)[b] in *benM*	[12]
pBAC887	Ap^R; Δ*catM51090*; PCR of ACIAD1444 [Fragment 1] (1,442,661-1,443,678)[b] with MTV1128 & MTV1129; *catB* [Fragment 2] (1,444,579-1,445,673)[b] with MTV1130 & MTV1131. DNA was digested with EcoRI+SmaI [Fragment 1] and SmaI+PstI [Fragment 2] and ligated to pUC18 cut with EcoRI+PstI.	This study
pBAC936	Ap^R; derived from pBAC7 by site directed mutagenesis to introduce a codon change [BenM(F18I); **TTC**(F) → **ATT**(I) in *benM*; (1,433,876-1,433,878)[b]] with primers MTV6 & MTV7.	This study

Table S2. continued

Plasmid	Relevant characteristics[a]	Source
pBAC937	Ap^R; *catM51095* [CatM(I18F), **A**TT (I)→**T**TT (F)] (1,444,540)[b]; *catM*5682 [CatM(F293Y), T**T**T (F)→T**A**T (Y)] (1,443,714)[b]]; DNA recovered from ACN737 by the gap-repair method [21] using linearized pBAC184	This study
pBAC938	Ap^R; Made by excising *catM51095* [CatM(I18F)] (1,444,540)[b] away from *catM5682* [CatM(F293Y)] as a NsiI+StuI fragment and ligating to pIB1 digested with the same enzymes.	This study
pBAC945	Ap^R; Made by excising *catM* as a XbaI+FspI fragment (1,443,168-1,445,025)[b] from pIB1 and ligating to pUC18 digested with XbaI+HincII.	This study
pBAC949	Ap^R; *ben* region (1,432,125-1,439,437)[b]; DNA recovered from ADP1 by the gap-repair method [21] using linearized pIGG13	This study
pBAC961	Ap^R; derived from pBAC945 by site directed mutagenesis to introduce a codon change [CatM(K38N), AA**A**(K) →AA**T**(N) in *catM*; (1,444,478)[b]] with primers MTV47 & MTV48.	This study
pBAC1025	Ap^R; *catM51234*; Made by SOEing PCR [22]; fragment 1 [*catM* (1,442,761-1,444,416)[b], portion encodes EBD-LH amplified with MTV3 & MTV76]; fragment 2 [*benM* (1,433,755-1,433,928)[b], portion encodes DBD amplified with MTV69 & MTV82]; fragment 3 [*catB* (1,444,591-1,445,673)[b], MTV81 & MTV1131. Fused fragment was digested with SacI+PstI and ligated to pUC18 digested with same enzymes.	This study
pBAC1027	Ap^R; Made by amplifying *catM51234* allele [$_{\text{BenM-DBD}}$CatM] with MTV63 & MTV66. Oligos add a 5′-NdeI and a 3′-XhoI to *catM51234*. Amplified region was digested with NdeI+XhoI and ligated to pET21-b digested with the same enzymes. Expression construct for $_{\text{BenM-DBD}}$CatM	This study
pBAC1040	Ap^R; derived from pBAC938 by site directed mutagenesis to introduce a codon change [CatM(K38N); AA**A**(K) →AA**T**(N) in *catM51095*; CatM(I18F); (1,444,478)[b]] with primers MTV47 & MTV48.	This study
pBAC1041	Ap^R; derived from pBAC936 by site directed mutagenesis to introduce a codon change [BenM(N38K); AA**T**(N) → AA**A**(K) in *benM51240*; BenM(F18I); (1,433,816)[b]]; with primers MTV43 & MTV44.	This study
pBAC1045	Ap^R; Made by amplifying *catM51249* allele [CatM(I18F,K38N)] with MTV65 & MTV66. Oligos add a 5′-NdeI and a 3′-XhoI to *catM51249*. Amplified region was digested with NdeI+XhoI and ligated to pET21-b digested with the same enzymes. Expression construct for CatM(I18F,K38N)	This study
pBAC1066	Ap^R; derived from pBAC1040 by site directed mutagenesis to introduce a codon change [CatM(F293Y); T**T**T(F) →T**A**T(Y) in *catM51249*; CatM(I18F,K38N); (1,443,714)[b]] with primers MTV1132 & MTV1133.	This study
pBAC1069	Ap^R; derived from pBAC1025 by site directed mutagenesis to introduce a codon change [CatM(F293Y); T**T**T(F) → T**A**T(Y) in *catM1234*; $_{\text{BenM-DBD}}$CatM; (1,443,714)[b] with primers MTV1132 & MTV1133.	This study

Table S2. continued

Plasmid	Relevant characteristics[a]	Source
pBAC1071	Ap^R; Site directed mutagenesis of pBAC1066 to change a codon [CatM(H160R); **CAT**(H) →**CGG**(R) in the *catM* allele encoding CatM(I18F,K38N,F293Y); (1,444,112-1,444,113)[b]] with primers MTV1134 & MTV1135	This study
pBAC1074	Ap^R; *benM51294*; Made by SOEing PCR [22]; fragment 1 [*benM,* (1,432,152-1,433,755)[b], portion encodes EBD-LH amplified with MTV94 & MTV97]; fragment 2 [*catM* (1,444,418-1,44,591)[b], portion encodes DBD amplified with MTV96 & MTV99]; fragment 3 [*benA* (1,433,930-1,434,954)[b], MTV95 & MTV98. Fused fragment was digested with SacI+PstI and ligated to pUC18 digested with same enzymes.	This study
pBAC1078	Ap^R; derived from pBAC1069 by site directed mutagenesis to introduce a codon change [CatM(H160R); **CAT**(H) →**CGG**(R) in *catM* allele encoding BenM-DBDCatM(F293Y); (1,443,714)[b] with primers MTV1134 & MTV1135.	This study
pBAC1086	Ap^R; Made by amplifying *catM51301* allele [BenM-DBDCatM(F293Y,H160R)] with MTV63 & MTV66. Oligos add a 5′-NdeI and a 3′-XhoI to *catM51301.* Amplified region was digested with NdeI+XhoI and ligated to pET21-b digested with the same enzymes. Expression construct for BenM-DBDCatM(F293Y,H160R)	This study
pBAC1085	Ap^R; Made by amplifying *catM51344* allele [CatM(I18F,K38N,H160R,F293Y)] with MTV65 & MTV66. Oligos add a 5′-NdeI and a 3′-XhoI to *catM51344.* Amplified region was digested with NdeI+XhoI and ligated to pET21-b digested with the same enzymes. Expression construct for CatM(I18F,K38N,H160R,F293Y).	This study
pBAC1108	Ap^R; derived from pBAC945 by site directed mutagenesis to introduce a codon change [CatM(F293Y); T**T**T(F) → T**A**T(Y) in *catM*; (1,443,714)[b] with primers MTV1132 & MTV1133.	This study
pBAC1109	Ap^R; derived from pBAC945 by site directed mutagenesis to introduce a codon change [CatM(H160R); **CAT**(H) →**CGG**(R) in *catM*; (1,443,714)[b] with primers MTV1134 & MTV1135.	This study
pBAC1140	Ap^R; derived from pBAC1108 by site directed mutagenesis to introduce a codon change [CatM(H160R); **CAT**(H) →**CGG**(R) in *catM5682* [CatM(F293Y)]; (1,443,714)[b] with primers MTV1134 & MTV1135.	This study

[a]Ap^R, ampicillin resistant; SmSp^R, streptomycin and spectinomycin resistant; Km^R, kanamycin resistant; ΩS omega cassette encoding SmSp^R and ΩK encoding Km^R [14,15]; *sacB*-Km^R, dual selection cassette containing a counterselectable sacB marker and Km^R cassette [17].
[b]Genomic positions in ADP1 (NCBI reference NC_005966)

Supplementary Table S3.

Table S3. Primers

Primers	Sequence (5′ → 3′)	Uses and Notes
MTV3	GAGTCA**GAGCTC**CGAGTTAAAGCGTC	Used to make BenM-DBDCatM (pBAC1025); with MTV76 amplifies 1653 bp of ACIAD1444-*catM*; **SacI** site
MTV6	TAATTTGTCTGCGGCTTTGGT**A**A**T**GCTTTGCTCCTCAACCAC	With MTV7 for **TTC** → **ATT** mutagenesis; codon change in *benM*; BenM(F18I)
MTV7	GTGGTTGAGGAGCAAAGCA**T**T**A**CCAAAGCCGCAGACAAATTA	With MTV6 for **TTC** → **ATT** mutagenesis; codon change in *benM*; BenM(F18I)
MTV12	CAAGATTTTGAATTTGTCGGC	For EMSA, with MTV26 amplifies P_{benA}
MTV26	GCTAGTATTAATGACGGGAAT	For EMSA, with MTV12 amplifies P_{benA}
MTV43	AATCCCCAATTCTTCTTCAAG**T**TTTTGAATTTGTCGGCTTAAGG	With MTV44 for AA**T** → **AAA** mutagenesis; codon change in *benM*; BenM(N38K)
MTV44	CCTTAAGCCGACAAATTCAAAA**A**CTTGAAGAAGAATTGGGGATT	With MTV43 for AA**T** → **AAA** mutagenesis; codon change in *benM*; BenM(N38K)
MTV47	ATTCTTCTTCGAGATTTTG**A**ATTTGTCGGCTGAGGGG	With MTV48 for AA**A** → **AAT** mutagenesis; codon change in *catM*; CatM(K38N)
MTV48	CCCCTCAGCCGACAAATTCAAAA**T**CTCGAAGAAGAAT	With MTV47 for AA**A** → **AAT** mutagenesis; codon change in *catM*; CatM(K38N)
MTV63	TCAATT**CATATG**GAACTTAGACATCTCCGC	Used with MTV64 to amplify *benM* and to introduce a **NdeI** site for cloning to pET21-b
MTV64	TCAATT**CTCGAG**CCAGTTTGGCGGCTCAGTAAA	Used with MTV63 to amplify *benM* and to introduce a **XhoI** site for cloning to pET21-b; removes stop codon
MTV65	TCAATT**CATATG**GAACTAAGACACCTCAGA	Used with MTV66 to amplify *catM* and to introduce a **NdeI** site for cloning to pET21-b
MTV66	TCAATT**CTCGAG**TTCGATGAGTGGCCTGATATG	Used with MTV65 to amplify *catM* and to introduce a **XhoI** site for cloning to pET21-b; removes stop codon
MTV69	CTGATAAAAAAACATGCCTGCTTCTGTTGTCTTGACCGGTCTGCT	Use to make BenM-DBDCatM (pBAC1025); with MTV82 amplifies 174 bp of *benM*; overlapping sequence (*catM*) for SOEing PCR [22]
MTV76	AGACCGGTCAAGACAACAGAAGCAGGCATGTTTTTTTATCAG	Use to make BenM-DBDCatM (pBAC1025); with MTV76 amplifies 1653 bp of ACIAD1444-*catM*; overlapping sequence (*benM*) for SOEing PCR [22]
MTV77	AACTTTTCAGCAGCTTTGGA	For EMSA, with MTV79 amplifies P_{catB}
MTV79	ACATTTAAAGGCGCCTTGAT	For EMSA, with MTV77 amplifies P_{catB}
MTV81	GCGGAGATGTCTAAGTTCCATTTATACGCCCTAATTGGT	Use to make BenM-DBDCatM (pBAC1025); with MTV1131 amplifies 1083 bp of *catB*; overlapping sequence (*benM*) for SOEing PCR [22]
MTV82	ACCAATTAGGGCGTATAAATGGAACTTAGACATTCTCCGC	Use to make BenM-DBDCatM (pBAC1025); with MTV82 amplifies 174 bp of *benM*; overlapping sequence (*catB*) for SOEing PCR [22]
MTV94	GAGTCA**GAGCTC**ATGGAAGTTTTGCCTGAATCGATTGAC	Use to make CatM-DBDBenM (pBAC1074); with MTV97 amplifies 1604 bp of *benK-benM*; **SacI** site
MTV95	GATGAT**CTGCAG**TGCCTGTTTTTCTTTACGATGCTG	Use to make CatM-DBDBenM (pBAC1074); with MTV98 amplifies 1604 bp of *benA*; **PstI** site
MTV96	GGCGTACTGATAAAAAAAGTGACCCTCAGGAGTCACTTTAGCCGGTCTGAA	Use to make CatM-DBDBenM (pBAC1074); with MTV99 amplifies 174 bp of *catM*; overlapping sequence (*benM*) for SOEing PCR [22]
MTV97	GGCTTCAGACCGAAAGTGACTCCTGAGGGTCACTTTTTTTATCAGTAC	Use to make CatM-DBDBenM (pBAC1074); with MTV94 amplifies 1604 bp of *benK-benM*; overlapping sequence (*catM*) for SOEing PCR [22]

Table S3. continued		
Primers	Sequence (5′ → 3′)	Uses and Notes
MTV98	CACAAAATATCTGAGGTGTCTTAGTTCCATT TAAAAATACTCCATAGG	Use to make CatM-DBDBenM (pBAC1074); with MTV95 amplifies 1604 bp of *benA*; overlapping sequence (*catM*) for SOEing PCR [22]
MTV99	TATAATAAAATACCTATGGAGTATTTTTAAA TGGAACTAAGACACCTC	Use to make CatM-DBDBenM (pBAC1074); with MTV96 amplifies 174 bp of *catM*; overlapping sequence (*benA*) for SOEing PCR [22]
MTV1128	CG**GAATTC**AGCGCTCACACAAAAATT	With MTV1129 amplifies 1653 bp of ACIAD1444 for deletion of *catM*; **EcoRI** site
MTV1129	TGC**CCCGGG**AATATGTCTGAAAAATT	With MTV1128 amplifies ACIAD1444 for deletion of *catM*; **SmaI** site
MTV1130	TG**CCCCGGG**TGTTCTTAGTTCCATTTA	With MTV1131 amplifies *catB* for deletion of *catM*; **SmaI** site
MTV1131	GATGAT**CTGCAG**CTCCAGTGTTTCAAAGG	With MTV1130 amplifies *catB* for deletion of *catM*; **PstI** site
MTV1132	GTGCGTTGCA**T**ACACCTCCTGGACACAG	With MTV1133 for TTT → TAT mutagenesis; codon change in *catM*; CatM(F293Y)
MTV1133	CTGTGTCCAGGAGGTGT**A**TGCAACGCAC	With MTV1132 for TTT → TAT mutagenesis; codon change in *catM*; CatM(F293Y)
MTV1134	GTTCTTT**CC**GCAACACGATACGTCGAATTG	With MTV1135 for CAT → CGG mutagenesis; codon change in *catM*; CatM(H160R)
MTV1135	CAATTCGACGTATCGTGTTGC**GG**AAAGAAC	With MTV1134 for CAT → CGG mutagenesis; codon change in *catM*; CatM(H160R)

References

1. Ruangprasert, A.; Craven, S.H.; Neidle, E.L.; Momany, C. Full-length structures of BenM and two variants reveal different oligomerization schemes for LysR-type transcriptional regulators. *J. Mol. Biol.* **2010**, *404*, 568-586.
2. Bundy, B.M.; Collier, L.S.; Hoover, T.R.; Neidle, E.L. Synergistic transcriptional activation by one regulatory protein in response to two metabolites. *Proc. Natl. Acad. Sci. U. S. A.* **2002**, *99*, 7693-7698.
3. Craven, S.H.; Ezezika, O.C.; Momany, C.; Neidle, E.L. LysR homologs in *Acinetobacter*: Insights into a diverse and prevalent family of transcriptional regulators. In *Acinetobacter Molecular Biology.*, Gerischer, U., Ed. Caister Academic Press: Norfolk UK, **2008**; pp. 163-202.
4. Schell, M.A. Molecular biology of the LysR family of transcriptional regulators. *Annu. Rev. Microbiol.* **1993**, *47*, 597-626.
5. Ezezika, O.C.; Haddad, S.; Clark, T.J.; Neidle, E.L.; Momany, C. Distinct effector-binding sites enable synergistic transcriptional activation by BenM, a LysR-type regulator. *J. Mol. Biol.* **2007**, *367*, 616-629.
6. Maddocks, S.; Oyston, P. Structure and function of the LysR-type transcriptional regulator (LTTR) family proteins. *Microbiology.* **2008**, *154*, 3609.
7. Ezezika, O.C.; Collier-Hyams, L.S.; Dale, H.A.; Burk, A.C.; Neidle, E.L. CatM regulation of the *benABCDE* operon: functional divergence of two LysR-type paralogs in *Acinetobacter baylyi* ADP1. *Appl. Environ. Microbiol.* **2006**, *72*, 1749-1758.
8. Juni, E.; Janik, A. Transformation of *Acinetobacter calcoaceticus* (Bacterium anitratum). *J. Bacteriol.* **1969**, *98*, 281-288.
9. Vaneechoutte, M.; Young, D.M.; Ornston, L.N.; De Baere, T.; Nemec, A.; Van Der Reijden, T.; Carr, E.; Tjernberg, I.; Dijkshoorn, L. Naturally transformable *Acinetobacter* sp. strain ADP1 belongs to the newly described species *Acinetobacter baylyi*. *Appl. Environ. Microbiol.* **2006**, *72*, 932-936.

10. Romero-Arroyo, C.E.; Schell, M.A.; Gaines, G.L., 3rd; Neidle, E.L. *catM* encodes a LysR-type transcriptional activator regulating catechol degradation in *Acinetobacter calcoaceticus*. *J. Bacteriol.* **1995**, *177*, 5891-5898.
11. Collier, L.S.; Gaines, G.L., 3rd; Neidle, E.L. Regulation of benzoate degradation in *Acinetobacter* sp. strain ADP1 by BenM, a LysR-type transcriptional activator. *J. Bacteriol.* **1998**, *180*, 2493-2501.
12. Craven, S.H.; Ezezika, O.C.; Haddad, S.; Hall, R.A.; Momany, C.; Neidle, E.L. Inducer responses of BenM, a LysR-type transcriptional regulator from *Acinetobacter baylyi* ADP1. *Mol. Microbiol.* **2009**, *72*, 881-894.
13. Seaton, S.C.; Elliott, K.T.; Cuff, L.E.; Laniohan, N.S.; Patel, P.R.; Neidle, E.L. Genome-wide selection for increased copy number in *Acinetobacter baylyi* ADP1: locus and context-dependent variation in gene amplification. *Mol. Microbiol.* **2012**, *83*, 520-535.
14. Eraso, J.M.; Kaplan, S. PrrA, a putative response regulator involved in oxygen regulation of photosynthesis gene expression in *Rhodobacter sphaeroides*. *J. Bacteriol.* **1994**, *176*, 32-43.
15. Prentki, P.; Krisch, H.M. In vitro insertional mutagenesis with a selectable DNA fragment. *Gene.* **1984**, *29*, 303-313.
16. Yanisch-Perron, C.; Vieira, J.; Messing, J. Improved M13 phage cloning vectors and host strains: nucleotide sequences of the M13mp18 and pUC19 vectors. *Gene* **1985**, *33*, 103-119.
17. Jones, R.M.; Williams, P.A. Mutational analysis of the critical bases involved in activation of the AreR-regulated sigma54-dependent promoter in *Acinetobacter* sp. strain ADP1. *Appl. Environ. Microbiol.* **2003**, *69*, 5627-5635.
18. Kokotek, W.; Lotz, W. Construction of a *lac*Z-kanamycin-resistance cassette, useful for site-directed mutagenesis and as a promoter probe. *Gene.* **1989**, *84*, 467-471.
19. Neidle, E.L.; Ornston, L.N. Cloning and expression of *Acinetobacter calcoaceticus* catechol 1,2-dioxygenase structural gene *catA* in *Escherichia coli*. *J. Bacteriol.* **1986**, *168*, 815-820.
20. Neidle, E.L.; Ornston, L.N. Benzoate and muconate, structurally dissimilar metabolites, induce expression of *catA* in *Acinetobacter calcoaceticus. J. Bacteriol.* **1987**, *169*, 414-415.
21. Gregg-Jolly, L.A.; Ornston, L.N. Recovery of DNA from the *Acinetobacter calcoaceticus* chromosome by gap repair. *J. Bacteriol.* **1990**, *172*, 6169-6172.
22. Horton, R.M.; Cai, Z.L.; Ho, S.N.; Pease, L.R. Gene splicing by overlap extension: tailor-made genes using the polymerase chain reaction. *Biotechniques.* **1990**, *8*, 528-535.
